# A threat to loyalty: Fear of missing out (FOMO) leads to reluctance to repeat current experiences

**DOI:** 10.1371/journal.pone.0232318

**Published:** 2020-04-30

**Authors:** Ceren Hayran, Lalin Anik, Zeynep Gürhan-Canli

**Affiliations:** 1 School of Business, Ozyegin University, Istanbul, Turkey; 2 Darden School of Business, University of Virginia, Charlottesville, Virginia, United States of America; 3 Graduate School of Business, Koc University, Istanbul, Turkey; University of Milan, ITALY

## Abstract

We investigate a popular but underresearched concept, the fear of missing out (FOMO), on desirable experiences of which an individual is aware, but in which they do not partake. Through laboratory and field studies, we establish FOMO’s pervasiveness as a psychological phenomenon, present real-life contexts wherein FOMO may be experienced, and explore its behavioral consequences. Specifically, we show that FOMO poses a threat to loyalty by decreasing one’s intentions to repeat a current experience and may decrease the valuation of the current experience.

## Introduction

Imagine that on a Friday evening, you are having dinner at a new restaurant you have been eager to try. As you are dining, your phone starts flashing with messages and social media notifications. Upon checking them, you become aware of other activities and experiences taking place in town: a new movie playing, a concert, a free pizza night, and numerous other events you did not know about. How would you feel? Would your awareness of alternative activities affect your dining experience that evening or your restaurant choice next time you dine? If you are a marketer, how would you appeal to the consumer in this moment?

Today, plagued by continual rushing and a sense of urgency, people pursue more, live faster, and feel that their resources are insufficient [1, 2]. Through digital tools, individuals have access to real-time information about experiences in their environment. Yet, since at any moment they can only be in one place, people end up missing many other experiences. Consequently, they are likely to experience an aversive feeling of missing out on known but unattended experiences, a concept known as “fear of missing out” (FOMO) [3, 4]. Our aim in this research is to explore this FOMO and its behavioral consequences. Importantly, while most of the scientific work to date has explored FOMO as a trait variable (i.e., an individual tendency to experience FOMO), we explore it as a momentary feeling that is situationally triggered upon learning about fleeting alternative experiences taking place in one’s environment.

### FOMO (fear of missing out)

FOMO entered the *Oxford English Dictionary* in 2013 [5] alongside other popular social media terminology like “selfie” and “emoji.” It stands for *“*fear of missing out,” and is defined as “anxiety that an exciting or interesting event may currently be happening elsewhere, often aroused by posts seen on social media.” Similarly, early academic work by Przybylski et al. [4] described FOMO as the “pervasive apprehension that others might be having rewarding experiences from which one is absent.” FOMO has become an increasingly popular social phenomenon with extensive media coverage [e.g., 6–8]. A recent hashtag tracking revealed that over 200 posts on Twitter and Instagram featured the #FOMO hashtag, reaching more than 100,000 individual views within a single day (www.keyhole.co). That same day, there were 182,000 FOMO-related YouTube videos. FOMO is so rampant in society that several digital applications have been developed to help consumers overcome it (e.g., FOMOsonar, NOMO—“no more missing out,” and Fomo).

Parallel to the media attention, there has been increasing academic interest in FOMO across diverse fields. Our review of the growing literature on FOMO by using “FOMO” and “fear of missing out” keywords presented twenty-one peer-reviewed articles on the correlates and consequences of FOMO in diverse scholarly fields (e.g., marketing, psychology, education and digital technologies). We provide a summary of current research findings on FOMO in Table 1. Most of the present research has explored FOMO as a trait variable in relation to usage of digital tools and social media. Przybylski et al. [4] have initially created a scale to measure individual differences in FOMO and demonstrated how FOMO is associated with increased negative mood, lower life satisfaction, and unmet psychological needs. In terms of its correlates, what we know so far is that some of FOMO’s common drivers include problematic internet use—specifically online social networks [15, 18]—and problematic mobile phone use [17, 19]. Follow-up studies associated FOMO with other important negative health outcomes such as stress [14, 24], anxiety, and depression [13, 22, 25]. FOMO’s consequences extend beyond negative effects and surface in different contexts; for example, studies in education revealed that FOMO negatively influences academic motivation [11], college adjustment [12], and study-abroad experiences [20]. In this paper, we aim to contribute to these growing efforts on FOMO’s consequences—specifically on behaviors.

**Table 1:** Summary of previous academic work on FOMO.

First author (year)	FOMO type explored	Method	Study topic
Abel (2016) [9]	trait FOMO	survey	Development of FOMO trait scale
Adams (2017) [10]	momentary FOMO	interview	Link between FOMO and poor sleep
Alt (2015) [11]	trait FOMO	survey	Link between FOMO and students’ academic motivation
Alt (2018) [12]	trait FOMO	survey	Link between FOMO and students’ maladjustment; SNS use
Baker (2016) [13]	trait FOMO	survey	Link between FOMO and need to belong; SNS use; negative health outcomes
Beyens (2016) [14]	trait FOMO	survey	Link between FOMO and SNS use; perceived stress; need to belong; need for popularity
Blackwell (2017) [15]	trait FOMO	survey	Link between FOMO and social media addiction
Buglass (2017) [16]	trait FOMO	survey	Link between FOMO and SNS use
Chotpitayasunondh (2016) [17]	trait FOMO	survey	Link between FOMO and phubbing; smartphone use
Dempsey (2019) [18]	trait FOMO	survey	Link between FOMO and problematic Facebook use
Elhai (2016) [19]	trait FOMO	survey	Link between FOMO and problematic smartphone use
Hetz (2015) [20]	trait FOMO	survey, focus group	Link between FOMO and students’ study-abroad experiences
Hodkinson (2016) [21]		focus group	Consumer behavior responses to FOMO-based advertising appeals
Hunt (2018) [22]	trait FOMO	experiment	Link between FOMO and SNS use
Lai (2016) [23]	trait FOMO	EEG recording	Neurobiological correlates of FOMO in response to social exclusion and social inclusion cues
Milyavskaya (2018) [24]	momentary FOMO	ESM, experiment	Assessment of the short and long-term consequences of experiencing FOMO; changes in momentary affective states; link with SNS use
Oberst (2016) [25]	trait FOMO	survey	Link between FOMO and psychopathological symptoms; SNS use
Przybylski (2013) [4]	trait FOMO	survey	Development of FOMO trait scale; link between FOMO and low life satisfaction, psychological need satisfaction, mood, and SNS use
Riordan (2015) [26]	trait FOMO	survey	Link between FOMO and alcohol use
Riordan (2018) [27]	trait FOMO	survey	Development of a single-item FOMO trait scale
Wang (2018) [28]	trait FOMO	survey	Link between FOMO and need to belong; SNS use

Recent work has started to explore FOMO as a momentary feeling, shedding light on when, why, and how it occurs. For example, Milyavskaya et al. [24] analyzed individuals’ momentary FOMO experiences in an experience-sampling study. They showed that FOMO mostly occurs later in the day and the week, and while one is doing a required task like studying or working. Adams [10] further showed that momentary FOMO leads to a delay in time when students go to sleep, because they do not want to miss out on socializing opportunities. Hodkinson [21] examined FOMO in the consumer context for the first time, and conceptually presented consumer response mechanisms to FOMO-based advertising appeals.

Our article extends prior work on FOMO in several ways: (1) While most of the previous work on FOMO treats it as a trait variable, we explore momentary FOMO triggered by information received at a particular moment, indicating a present-time orientation. Hence, regardless of individuals’ propensity to experience FOMO, we examine FOMO that occurs situationally in response to contextual factors. (2) We explore FOMO’s behavioral consequences and provide managerial implications. Specifically, we show that FOMO decreases one’s intentions to repeat a current experience and may also negatively influence the valuation of the current experience, which may pose a threat to loyalty. (3) While previous research predominantly relied on using surveys to identify FOMO’s emotional and behavioral correlates, we use experiments to identify cause-and-effect relationships. Through lab and field experiments, we aim to uncover real-life contexts wherein FOMO may occur and understand realistic responses to FOMO. (4) Finally, we provide a detailed conceptual discussion on FOMO’s meaning in a nomological web of constructs. Theoretically and empirically, we distinguish FOMO from regret, anticipated regret, envy, and social exclusion, and argue that, especially when an individual is involved in an enjoyable experience, these negative emotions do not necessarily accompany FOMO.

The rest of our article is organized as follows. First, we provide a theoretical discussion on FOMO’s distinction from other psychological constructs. Then, we provide two hypotheses on FOMO’s behavioral consequences and present four studies to test these. Lastly, we discuss the theoretical and managerial implications of our findings, address the limitations of our work, and suggest future research directions.

### Differentiating FOMO from other psychological constructs

Research to date has not explained FOMO through the lens of a specific theory. To understand what FOMO–as a momentary feeling–is, it is important to know how it is different from seemingly related constructs of regret, anticipated regret, envy, and social exclusion. Next, we theoretically elaborate on FOMO’s distinction from these concepts.

#### Regret and anticipated regret

It could be argued that FOMO has a similar psychological process to regret and anticipated regret. Regret refers to the negative evaluation of a past decision [29]. It motivates people to think about counterfactuals—i.e., what different outcomes would have occurred had they decided differently. Anticipated regret, on the other hand, is the negative feeling resulting from imagining future regret before making a decision [30]. Hence, individuals experience regret about past decisions and anticipated regret in relation to potential future decisions.

We argue that FOMO is distinct from regret and anticipated regret in several aspects. First, these three constructs signify different time orientations. FOMO entails a present-time orientation about one’s current situation, whereas regret is a retrospective feeling about past decisions and anticipated regret is a prospective feeling about as-yet-unmade decisions.

Second, regret and anticipated regret result from a person’s own actions or inactions, thus incorporating responsibility and self-blame. Personal agency differentiates regret and anticipated regret from other negative emotions such as anger, anxiety, fear, or disappointment [31]. FOMO, however, does not always involve personal liability. An individual may learn about attractive alternative options while already engaged in an activity and experience FOMO momentarily. In such a case, since the alternatives were unknown at the time of the decision to participate in the current activity or attending the alternatives is not currently possible, feelings of responsibility and self-blame should not occur. For example, a person may experience FOMO upon seeing the queue outside H&M for the invite-only Moschino collection first-day sale, as she could not be among the early buyers. Since the consumer was not invited and did not know about the sale, there would not be a past decision to regret. Because attending the sales event is no longer possible, there cannot be anticipated regret either. As this example reveals, being aware of an ongoing desirable experience may induce FOMO, yet may not create regret about a past decision or anticipated regret regarding a future decision. Nevertheless, if the current FOMO results from a deliberate decision made in the past (e.g., the person was invited but preferred not to go), regret may accompany FOMO. Similarly, if attending the alternative experience is currently possible, anticipated regret may be experienced along with FOMO. Therefore, we argue that FOMO occurs independently of regret and anticipated regret, but may be accompanied by these emotions.

Third, regret requires the realization that another decision would have been better, and is therefore experienced if a decision’s outcome is negative [31]. As we show in study 1C, FOMO may be experienced even during experiences individuals find highly enjoyable and have no negative emotions about. Mere awareness of the desirable alternatives may induce FOMO, even though a person may be content with the current situation.

#### Envy

Envy arises from someone else’s superior achievement, success, advantages, or possessions [32]. It is a detrimental emotion that may decrease well-being and result in willingness to reduce the perceived gap with the envied person, sometimes at the expense of harming that person [33]. Envy is also explained as a feeling of admiration with a component of hostility [34]. It occurs as a result of comparing one’s situation with others’, especially when making upward comparisons [35] or, more intensely, when making comparisons with similar others [36].

The main distinction between FOMO and envy is that envy necessitates a target person, while FOMO might simply result from awareness of unattended attractive experiences. If FOMO is experienced upon learning about others’ experiences and induces a comparison of one’s situation with others’, then envy may accompany FOMO. Yet FOMO may be experienced upon learning about any kind of desirable event or activity (e.g., a sales event) without specific knowledge of others’ involvement. Therefore, we argue that FOMO is an emotion distinct from envy, despite envy sometimes accompanying FOMO.

#### Social exclusion

Social exclusion refers to being excluded or left alone by society or by a reference group [37]. Social connection is a fundamental human need, and feeling excluded may cause detrimental psychological, emotional, and behavioral consequences. When individuals feel excluded, they may become motivated to affiliate with others to restore their belongingness in several ways [38]) such as by regaining group membership [39], by conforming to others, by mimicking others [40], or by consuming products that are symbolic of their reference groups [41].

Although deliberate social exclusion might induce FOMO, it is not a necessary condition. FOMO often occurs when an individual becomes aware of unattended experiences without other individuals’ deliberate intention. As with envy, feeling socially excluded may or may not accompany FOMO. For example, a person may experience both FOMO and social exclusion upon seeing photos of a friend’s party that she was not invited to. However, one may experience FOMO without feeling excluded, such as upon reading the news about the release of Game of Thrones’ final episode while working at the office. It is possible that news about an ongoing event or activity might trigger thoughts about others’ involvement, but exclusion is not a necessary ingredient for FOMO.

In sum, we argue that although FOMO may relate to constructs such as regret, anticipated regret, envy, and social exclusion, it is still a distinct emotion that can be experienced in the absence of seemingly related affective experiences. Next, we discuss FOMO’s consequences and present our hypotheses.

### The current research

In this digital era, at any moment, individuals can easily reach information about what is happening around them, and what they are missing out on. Knowledge about existing attractive alternatives has been shown to impact commitment decisions in professional and interpersonal contexts in different research streams. For example, in the consumer context, knowledge about viable competing alternatives (e.g., hairstyling or banking services) is likely to increase switching intentions to other service providers [42, 43]. In organizations, availability of competing alternatives may decrease employees’ job commitment [44]. Likewise, interpersonally, becoming aware of attractive alternative romantic partners may decrease commitment to a current relationship [45, 46]. These results suggest that when making commitment decisions, individuals may compare what is at hand with available alternatives, which might influence their intentions to stay or to leave the status quo.

We build on these findings by exploring how momentary FOMO, which occurs as a result of learning about fleeting alternative experiences, affects intentions to repeat current experiences. We predict that, when already involved in an activity, experiencing FOMO upon learning about other attractive events and experiences motivates pursuit of the alternatives rather than repeated involvement in the current activity. In other words, we propose that FOMO negatively influences individuals’ likelihood to repeat their current experience based on the salience of the missed opportunities. Hence, we extend previous research findings on the effects of alternative attractiveness on commitment judgments by exploring momentary experiences through the lens of FOMO. Importantly, we show that individuals may experience FOMO and a reluctance to repeat their current experiences even when controlling for their variety-seeking tendencies and when their enjoyment of the current activity is high. More formally stated.

**H1:** Experiencing FOMO momentarily decreases intentions to repeat a current activity (i.e., redo or revisit intentions).

People have the tendency to focus on the forgone options [47], hence they see the unrealized alternatives in a positive light. In evaluating one’s circumstances, what is forgone becomes particularly salient and attractive in memory. Supporting this notion, research on memory has established that increasing the accessibility of alternatives in memory is likely to increase their consideration [48]. FOMO indicates that exposure to information about attractive alternatives is a reminder that every activity attended means (at least) another event missed. We argue that mere awareness of the missed opportunities may lead one to perceive alternatives in a positive light, relatively leading to lower valuation of one’s current situation. In other words, we predict that becoming aware of unattended attractive activities taking place in one’s environment at a particular moment may decrease the valuation of one’s current circumstances. Specifically, we show that this may occur even during enjoyable experiences when one is satisfied with their current situation. More formally stated.

**H2:** Experiencing FOMO momentarily decreases the valuation of current experiences.

## Materials and methods

Ethical approval: Data collection for the experiments were approved by the Koc University Social Sciences Research Ethics Committee (2015.012IRB3.007), and Duke University IRB Committee (C0971). Written informed consent was obtained for all studies.

Next, we present an exploratory Amazon Mechanical Turk (MTurk) study that establishes FOMO’s pervasiveness among individuals: one lab and two field experiments that test the hypotheses in diverse real-life situations. In all studies, FOMO is induced by creating awareness of concurrent, unattended experiences. Previous academic studies on FOMO reveal that it is mostly experienced when doing a required task such as working and studying [24], and that it is pervasive among university students [e.g., 11, 14, 24]. Press articles also suggest that individuals frequently experience FOMO when they learn about presumably more enjoyable activities taking place while they are working or studying [49, 50]. As suggested by these real-life cases and the previous academic evidence, we explore “summertime FOMO” and “workplace FOMO” in studies 1A and 1B, respectively. In study 1C, we expand our investigation to explore the same hypothesized effects when individuals are involved in highly enjoyable experiences, specifically during an entertaining after-work event.

### Exploratory study

First, we conducted a short exploratory study to understand FOMO’s pervasiveness and how frequently individuals experience it. We recruited 936 individuals on the MTurk online panel (*M*_age_ = 33.20, *SD* = 13.58, 45% women). They read a detailed description of FOMO and indicated whether they experience FOMO in general (1 = yes, 2 = no), and to what extent (1 = never, 7 = always; please see S1 Appendix for the scenario). Fifty-eight percent of the participants indicated experiencing FOMO in general, and 81% indicated experiencing it at least occasionally. These results suggest that FOMO is a commonly experienced affective state.

### Study 1A—Summer school lab study

Study 1A explores H1, specifically whether FOMO decreases people’s intentions to repeat a current activity, in a controlled lab setting. As suggested by media coverage, we predicted that college students are likely to experience FOMO upon learning that while they are attending school during, for example, spring or summer break, other students are engaged in enjoyable experiences. Specifically, we examine whether learning about peers’ vacation experiences induces FOMO for students attending summer school, and whether this decreases students’ intentions to reattend summer school the following year. To test this realistically, we conducted study 1A with students attending summer school classes at a university.

We induced FOMO with a scenario summarizing the results of a hypothetical survey conducted with college students about how they preferred to spend their summer vacations. We first ran a pretest to identify students’ highly favored summer vacation destinations, which we used as stimuli in the main study. Fifty undergraduates from the same university, with similar demographics to the target group (*M*_age_ = 21.6, *SD* = 1.19, 55% women) were randomly approached on campus and asked to indicate summer vacation spots that they liked the most and viewed as self-relevant. A total of 19 destinations were mentioned, including 11 cities within 8 different countries. From the responses, the most-mentioned 4 countries and 4 cities were selected to be used within the main study’s manipulation scenario (see S1 Appendix for stimuli used in study 1A).

#### Method

A total of 83 summer school students (*M*_age_ = 21.4, *SD* = 1.68, 58% female) who were enrolled in a marketing course participated in the study in exchange for course credit, and were randomly assigned to either the control or FOMO condition. Students in the FOMO condition read about how other students were spending their summer vacation. The scenario presented students’ preferred cities and countries to visit that summer, as revealed by the pretest, along with vacation photos. The students in the control condition read general information about the university (e.g., history and a brief program overview), which was presented alongside campus photos. We tried to keep the scenarios’ length and visual qualities parallel in both conditions. As a manipulation check, participants were explicitly asked to indicate the extent to which they experienced FOMO, as in Milyavskaya et al. [24] (“In this moment, to what extent do you feel like you are missing out on alternative activities and experiences taking place in your environment?”; 1 = not at all, 7 = very much). Participants also indicated their intentions to attend summer school again the following year: “How much are you willing to stay for summer school next year?”; 1 = not at all, 7 = very much.” Given that 30% of the student body at this university attends summer school every summer and many of them are repeat attendees, the intention to attend summer school again was a realistic measure.”

It is possible that individuals’ variety-seeking tendencies, which correspond to the degree to which they express a desire to try new and different things, can influence repeat behaviors [51]. To make sure that variety-seeking as a trait variable did not influence students’ intentions to reattend summer school, we measured variety-seeking tendencies with three items (“When I go to a restaurant I feel it is safer to order dishes I am familiar with”; “If I like a brand, I rarely switch from it just to try something different”; “I enjoy taking chances in buying unfamiliar brands just to get some variety in my purchases”; 1 = strongly disagree, 7 = strongly agree; α = .67; [51]) and controlled for this variable in our analyses.

Finally, to make sure that our manipulation did not induce other affective states, and that FOMO drove the hypothesized effects, participants responded to measures of regret (“How much do you regret attending summer school?”), anticipated regret (“How much regret will you experience in the future for not being involved in other activities currently?”; [52]), anxiety (“I feel anxious”), and feelings of exclusion (“I feel excluded”; [38]), all on a seven-point scale (1 = not at all, 7 = very much).

#### Results and discussion

*Manipulation check*. As expected, the FOMO condition reported experiencing stronger FOMO (*M*_*FOMO*_ = 4.68, *SD* = 1.64) than the control condition (*M*_*control*_ = 3.71, *SD* = 1.92), *F*(1, 81) = 6.13, *p* = .02. Hence, summer school students experienced a higher level of FOMO when they read about other students’ ongoing vacation experiences than when they read factual information about the university’s history.

*Redo intentions*. A one-way ANOVA revealed that compared to the control condition, the FOMO condition was less likely to attend summer school again (*M*_*FOMO*_ = 4.05, *SD* = 2.17) vs. (*M*_*control*_ = 5.17, *SD* = 2.20), *F*(1, 81) = 5.44, *p* = .02. However, there were no significant differences between the two conditions in terms of participants’ feelings of anticipated regret (*M*_*FOMO*_ = 3.27, *SD* = 1.96 vs. *M*_*control*_ = 3.69, *SD* = 1.87), anxiety (*M*_*FOMO*_ = 3.12, *SD* = 1.78 vs. *M*_*control*_ = 3.52, *SD* = 2.09), or exclusion (*M*_*FOMO*_ = 3.20, *SD* = 1.94 vs. *M*_*control*_ = 2.71, *SD* = 1.63); all *F*s < 1.6, all *p*s > .21. Most importantly, there were no differences in participants’ feelings of regret for being in summer school (*M*_*FOMO*_ = 2.17, *SD* = 1.32) vs. (*M*_*control*_ = 1.83, *SD* = 1.12), *F*(1, 81) = 1.57, *p* = .21, which shows that although they experienced FOMO, students didn’t regret their choice to attend summer school upon learning about their peers’ enjoyable vacation experiences. When we controlled for affective states and participants’ variety-seeking tendencies, our results remained significant. Specifically, a one-way ANCOVA with regret, anticipated regret, anxiety, and exclusion as the covariates revealed that compared to the control condition, the FOMO condition was less likely to attend summer school again, *F*(1, 77) = 4.59, *p* = .04. Further, an ANCOVA with variety-seeking tendencies as the covariate did not change the results’ significance, *F*(1, 80) = 6.05, *p* = .02. Summary of mean ratings for FOMO and intentions to repeat the current activity across studies are presented in Table 2.

**Table 2:** Summary of mean ratings for experienced FOMO and intentions to redo the current activity.

	Study 1A Summer School		Study 1B Office		Study 1C Museum Event	
	FOMO	Control	FOMO	Control	FOMO	Control
Level of FOMO	4.68	3.71	5.38	4.63	4.09	3.21
Redo Intentions	4.05	5.17	3.54	4.49	5.48	6.12

Scales range from 1 (not at all) to 7 (very much).

*Discussion*. Study 1A provides initial support for the prediction that FOMO decreases individuals’ willingness to repeat the current activity in the near future (H1). Next, study 1B aims to replicate this finding in a field experiment by using another real-life setting; during overtime work at the office.

### Study 1B—Office field study

In line with previous research and press articles, we predicted that overtime at the office is another context wherein FOMO may easily occur. Therefore, we investigated whether FOMO similarly influences employees’ likelihood to work overtime again. As an additional supporting DV, we also explored how FOMO influences employees’ momentary job satisfaction. To test these realistically, we conducted an experiment with employees from six different companies. We induced FOMO by informing participants about the after-work events concurrently taking place in the city while they were working overtime at the office.

We first ran a pretest to select the stimuli for the main study. Thirty individuals with similar demographics to the target group (*M*_age_ = 32.73, *SD* = 4.34, 60% women) read brief descriptions and saw pictures of 10 after-work events taken from a widely used event website in the country (www.biletix.com). To make sure that the events we selected would be desirable and relevant, we asked participants to answer four questions (on a 7-point scale; 1 = not at all, 7 = very much): two questions about each event’s favorability—“How favorable do you think this event would be?” and “How desirable do you think this event is?” (minimum α = .92)—and two questions about how relevant they found the events—“To what extent can you picture yourself attending this event?” and “To what extent does this event relate to your life and your experiences?” (minimum α = .88). We used the six activities that received the highest ratings on both favorability and self-relevance in the main study (see Table 3 for ratings and the S1 Appendix for the final stimuli used in study 1B).

**Table 3:** Summary of mean ratings for stimuli used in study 1B and 1C pretests.

	Study 1B (*N* = 30)		Study 1C (*N* = 55)	
	Favorability	Self-relevance	Favorability	Self-relevance
Event 1	4.74	4.23	4.25	3.94
Event 2	5.17	4.85	4.44	3.75
Event 3	5.38	5.25	4.61	4.03
Event 4	4.75	4.25	4.42	3.44
Event 5	5.32	4.97	4.47	4.15
Event 6	4.80	4.52	4.35	3.50
Event 7*	4.47	4.20	4.13	3.28
Event 8*	4.47	4.22	3.45	2.99
Event 9*	4.53	4.12		
Event 10*	3.40	2.75		

Events marked with * indicate stimuli included in the pretest but not selected for the main study.

#### Method

We conducted the study with employees of six different companies: two telecommunications companies, two consulting firms, an advertising agency, and an e-commerce start-up. Participants were recruited through the researchers’ own network of friends, hence convenience sampling was used. Seventy-four individuals who were working at the office after 18:00 participated in our study (*M*_age_ = 30.1, *SD* = 4.51, 57% women). Participants were typically expected to work 45 hours a week, from 8:00/9:00 to 17:00/18:00, and all indicated working overtime at least occasionally. Participation was voluntary, and participants who successfully completed the study were entered into a lottery to win one of five pairs of movie tickets. The experiment was conducted in the local language to make the stimuli realistic and to prevent any language-related barriers in collecting data.

Participants were randomly assigned to the FOMO or control condition. Participants in the FOMO condition read information about six events that were concurrently taking place in town, which were presented along with real event photographs. Those in the control condition read neutral information about the city’s geopolitical importance, illustrated with landscape photographs. As a manipulation check, participants were asked to indicate the extent to which they experienced FOMO with the following statements: “It bothers me that I am in the know about but out of touch with the activities and experiences that are going on around”; “I am worried that I can’t take part in the activities that are going on in my surroundings” (1 = not at all true of me, 7 = very true of me); “In this moment, to what extent do you feel like you are missing out on alternative activities and experiences taking place in your environment?” (1 = not at all, 7 = very much; α = .88). For dependent variables, we measured participants’ willingness to work overtime the following week (“How much would you be willing to stay for overtime work next week if your boss asks you to?”) and momentary job satisfaction (“How satisfied do you feel with your job at the present moment?”; 1 = not at all, 7 = very much). To make sure that FOMO rather than any other affective states drove the hypothesized effects, we measured anxiety, exclusion, and variety-seeking tendencies, as well as envy (“I feel envious of other people”; 1 = not at all, 7 = very much; [32]) and mood (“I am in a 1 = very negative to 7 = very positive mood right now”). These measures were used as covariates in the analyses.

One potential explanation we have not yet addressed is that people who experience FOMO could perceive that there are more alternative activities concurrently taking place than those in the control condition. In order to capture this, we asked participants to guess how many events could be going on in the city that evening; their responses were used as a covariate (4-point scale: 1 = fewer than 10 events, 2 = 10–20 events, 3 = 21–30 events, 4 = more than 30 events). Importantly, this question was asked after the manipulation question, to ensure that participants in the FOMO (vs. control) condition didn’t imagine a higher number of social activities taking place in their environment.

#### Results and discussion

*Manipulation check*. As expected, participants in the FOMO condition experienced stronger FOMO (*M*_*FOMO*_ = 5.38, *SD* = 1.32) than those in the control condition (*M*_*control*_ = 4.63, *SD* = 1.44), *F*(1, 71) = 5.37, *p* = .02.

*Redo intentions and momentary job satisfaction*. A one-way ANOVA revealed that participants in the FOMO condition indicated lower willingness to work overtime again the following week (*M*_*FOMO*_ = 3.54, *SD* = 1.88 vs. *M*_*control*_ = 4.49, *SD* = 1.24), *F*(1,72) = 6.53, *p* = .01, and lower momentary job satisfaction (*M*_*FOMO*_ = 3.65, *SD* = 1.44 vs. *M*_*control*_ = 4.32, *SD* = 1.16), *F*(1,72) = 4.96; *p* = .03. There were no significant differences between the two conditions in terms of feelings of anxiety (*M*_*FOMO*_ = 4.40, *SD* = 1.61 vs. *M*_*control*_ = 3.94, *SD* = 1.52), envy (*M*_*FOMO*_ = 3.34, *SD* = 1.63 vs. *M*_*control*_ = 2.85, *SD* = 1.48), exclusion (*M*_*FOMO*_ = 2.14, *SD* = 1.35 *M*_*control*_ = 2.21, *SD* = 1.23), or mood (*M*_*FOMO*_ = 4.06, *SD* = 1.32 vs. *M*_*control*_ = 4.26, *SD* = 1.25)—all *F*s < 1.72, all *p*s > .19. The results remained significant when we controlled for affective states and variety-seeking tendencies. Specifically, a one-way ANCOVA revealed that when anxiety, exclusion, envy, and mood were controlled for, those in the FOMO condition were still less willing to work overtime again the following week, *F*(1, 63) = 4.51, *p* = .04; they also had lower job satisfaction momentarily, *F*(1, 63) = 4.78, *p* = .03. Further, when variety-seeking tendencies were controlled for, our results remained significant in terms of willingness to work overtime again the following week, *F*(1, 65) = 5.33, *p* = .02, and momentary job satisfaction, *F*(1, 65) = 6.30, *p* = .02.

Finally, we found that participants in the FOMO and control conditions didn’t significantly differ in terms of the number of alternative events they thought were going on in their environment; about 21–30 captured by (*M*_*FOMO*_ = 3.09, *SD* = 1.00) vs. (*M*_control_ = 3.29, *SD* = .87), *F*(1,66) = .82; *p* = .34. When we conducted a one-way ANCOVA with participants’ guesses about the number of existing events as the control variable, our results still remained significant in terms of participants’ willingness to work overtime again the following week, *F*(1, 65) = 7.61, *p* = .01), and their momentary job satisfaction, *F*(1, 65) = 6.68, *p* = .01.

*Discussion*. Study 1B provides further support for H1, demonstrating another setting wherein FOMO occurs. Additionally, this study suggests that FOMO may impair judgments of current experiences by showing reduced momentary job satisfaction upon experiencing FOMO. The results of studies 1 and 2 contribute to our understanding of FOMO’s consequences by showing that FOMO decreased individuals’ willingness to repeat the current activity in the near future. Yet, these results are confined to experiences where individuals are involved in rather constraining required activities. Differently, study 3 explores the hypothesized effects during highly enjoyable experiences.

### Study 1C—Museum field study

Studies 1A and 1B explored FOMO realistically among individuals involved in potentially not-so-fun activities (attending summer school and working overtime). In our final study, we expand our exploration in two ways. First, we investigate whether people can experience FOMO during highly enjoyable experiences and whether it leads to the same hypothesized effects. Second, in addition to testing individuals’ intentions to repeat the current activity (H1), we examine whether FOMO influences how consumers evaluate their current experience (H2).

To select the main study stimuli, we ran a similar pretest to the one presented in study 1B. A total of 55 participants from MTurk’s online panel (*M*_age_ = 37.13, *SD* = 13.16, 53% women) read brief descriptions and saw pictures of eight typical city events and rated them in terms of favorability and self-relevance (see Table 3 for ratings). We picked the six top-rated activities for the main study (see S1 Appendix for stimuli used in study 1C).

#### Method

We conducted an experiment during an after-work 21-and-up social event at a southeastern U.S. science museum. The entrance fee was $24.00 and all 400 available tickets had been sold. During the event, visitors could sample beer and participate in interactive games across 26 different stations. We had a table where visitors could fill out a five-minute survey for a chance to win a free museum membership for the whole year. Participants completed the survey either on iPads or on papers provided to them.

Sixty-seven participants (*M*_age_ = 29.51, *SD* = 6.02, 46% female) were randomly assigned to FOMO or control conditions. As expected, mean enjoyment level during the event was high (*M*_*enjoyment*_ = 6.34, *SD* = .69) 1 = not at all enjoyable, 7 = very much enjoyable). While those in the FOMO condition read information about six events concurrently taking place in town, control condition participants were given neutral information about local landmarks. As a manipulation check, participants indicated the extent to which they experienced FOMO (“In this moment, to what extent do you feel like you are missing out on alternative activities and experiences taking place in your environment?,” 1 = not at all, 7 = very much). Intention to revisit the museum (H1) was measured by first asking participants an explicit question about their revisit intentions—“How likely are you to come to the next museum event (vs. attend an alternative event)? (1 = not at all, 7 = very likely)”—and then asking them to choose which free museum membership they would prefer if they won our lottery: a 12-month free membership to the same museum in which the event was taking place or a 4-month free membership to four different area museums. This question tested whether the visitors were interested in repeating an experience similar to the current one or were motivated to seek other experiences. We expected to find that those in the FOMO condition would prefer the 4-month free membership to four different museums in the area more than those in the control condition. Next, we also tested whether FOMO negatively influenced individuals’ valuations of their current experience (H2) by asking how much they would be willing to accept (WTA on a $0–$40 sliding scale) to quit their current activity and attend an alternative activity. Finally, we measured variety-seeking tendencies and feelings of regret by asking participants for their guesses about how many events could be going on in the area that evening. We used these measures as covariates.

#### Results and discussion

*Manipulation check*. As expected, participants in the FOMO condition experienced stronger FOMO (*M*_*FOMO*_ = 4.09, *SD* = 1.61) than those in the control condition (*M*_*control*_ = 3.21, *SD* = 1.90), *F*(1, 65) = 4.22, *p* = .04.

*Redo intentions and WTA*. A one-way ANOVA revealed that participants in the FOMO condition were less likely (*M*_*FOMO*_ = 5.48, *SD* = 1.21) than those in the control condition (*M*_*control*_ = 6.12, *SD* = .91; *F*(1, 65) = 6.43, *p* = .01) to attend the next museum event, and more likely to attend an alternative event. Importantly, these results remained significant when we controlled for variety-seeking tendencies, which could influence revisit intentions. A one-way ANCOVA with variety-seeking tendencies as the covariate revealed that those in the FOMO condition were less likely than those in the control condition to come to the next museum event, *F*(1, 64) = 6.33, *p* = .01. There were no differences in feelings of regret between the FOMO (*M* = 1.42, *SD* = .48) and control conditions (*M* = 1.21, *SD* = .71), *F*(1, 65) = 2.20, *p* = .14, suggesting that the museum event participants did not regret being involved in their current activity upon learning about other attractive events. A one-way ANCOVA with regret as the covariate again revealed that those in the FOMO condition were less likely than those in the control condition to come to the next museum event, *F*(1, 64) = 4.53, *p* = .04. Furthermore, participants’ guesses about the number of area events that evening did not differ between the FOMO (*M* = 15.52, *SD* = 12.32) and control conditions (*M* = 14.62, *SD* = 10.76), *F*(1, 65) = .10, *p* = .75. The difference between the two conditions in terms of museum revisit intentions remained significant when we controlled for event count *F*(1, 64) = 6.23, *p* = .02.

In addition, a chi-square analysis revealed that people in the FOMO condition preferred the free membership to four different museums (as opposed to a free 12-month membership to the same museum) to a higher extent than those in the control condition (60% vs. 41%, *X*^*2*^(1) = 4.75, *p* = .03), supporting the hypothesis that FOMO decreased revisit intentions.

Finally, participants in the FOMO condition were more willing (*M*_*FOMO*_ = $22.0, *SD* = 11.84) than those in the control condition (*M*_*control*_ = $28.3, *SD* = 9.75; *F*(1, 65) = 5.66, *p* = .02) to accept a lower dollar amount to leave the event and attend an alternative activity. The WTA in the FOMO condition was lower than the event’s entrance fee ($24), suggesting that FOMO might even lead to incurring costs. The results remained significant when we controlled for variety-seeking tendencies (*F*(1, 64) = 5.73, *p* = .02) and guesses about the number of other events taking place (*F*(1, 65) = 5.51, *p* = .02), and only became marginally significant when controlling for feelings of regret (*F*(1, 65) = 3.63, *p* = .06). These results suggest that in this setting, regret might have had some effect on participants’ revisit intentions, but not enough to explain the full effect, suggesting that FOMO still plays a distinct role in individuals’ preferences.

*Discussion*. Study 1C provides further evidence for H1 in a third and unique setting, and shows that people may experience FOMO even during highly enjoyable experiences. This study also supports H2 by demonstrating that FOMO may lead people to value their current experiences less, and may motivate switching intentions away from the current experience.

## General discussion

### Theoretical and managerial implications

With increased reliance on digital technologies and use of social networking platforms that enable real-time information flow, FOMO has attracted attention in both academia and the marketplace. Responding to calls for further exploration of FOMO [3, 4], in this research, we examined FOMO as a momentary feeling in a nomological web of constructs, identified its consequences on behavior, and presented real-life contexts wherein FOMO may be experienced.

Our work contributes to the growing academic work on FOMO by suggesting novel behavioral consequences. While previous work primarily examined FOMO as a trait variable, we explored it as a momentary feeling that is triggered by information received at a particular moment. Such exploration allows us to both study FOMO in a broader population and to identify how it situationally affects decisions and behaviors. Specifically, across a series of laboratory and field experiments, we demonstrated that experiencing FOMO decreases valuations of and intentions to repeat current experiences, thereby posing a threat to loyalty.

Moreover, our work extends previous findings on how knowledge of attractive alternatives negatively influences commitment decisions in diverse professional and personal contexts [e.g., 42, 45]. We show that experiencing FOMO upon learning about other attractive activities decreases individuals’ intentions to repeat a current activity. Hence, we suggest that experiencing FOMO may be another means of mitigating commitment intentions.

Most of the existing research has associated FOMO with usage of digital tools and social media. It is established that social media usage is highly likely to evoke experiences of FOMO by reminding people about unattended alternative experiences in real time. However, FOMO is not restricted to online experiences [24]. Contributing to this early work, we focus on how individuals feel FOMO in diverse offline contexts such as the office, summer school, and a museum, which make our results more generalizable to other contexts wherein people might experience FOMO.

Although not in the main scope of our work, both conceptually and empirically, our work shows that FOMO is a distinct feeling from the seemingly related constructs of regret, anticipated regret, envy, and social exclusion. While such negative feelings may occur along with FOMO, they don’t have to be present for FOMO to be experienced. Most importantly, we show that FOMO may even be experienced during highly enjoyable experiences (e.g., a fun social event) where these negative feelings are not present. Examining FOMO’s interplay with these negative feelings in more detail would be an issue of theoretical importance for future research.

Our studies have important implications for managers. FOMO as a psychological phenomenon and social media buzzword is driving companies to explore its implications. Our results suggest that awareness of missed opportunities can momentarily induce FOMO and a related devaluation of and reluctance to repeat one’s current behavior. We show that this may occur both during somewhat negative experiences as well as during more favorable and entertaining events. Specifically, while working overtime at the office or taking courses during the summer, individuals can learn about desirable activities taking place in their environment. If managers or instructors know about the psychology of FOMO, they can help employees and students combat it by, for example, designing a more enjoyable work/study environment, providing incentives to increase motivation and appreciate the work at hand, and limiting or helping individuals avoid social media use during work time. Such practices may help individuals see their current experience in a more positive light, and thereby avoid FOMO’s negative effects.

### Limitations and future directions

Our work has some limitations. To the best of our knowledge, this article is the first to explore FOMO’s effects in real-life contexts; with one controlled lab experiment and two field experiments. Field experiments have high representativeness, i.e., behavior in a field experiment is very likely to reflect real life because of its natural setting; [53]. Accordingly, we tried to explore FOMO’s outcomes in natural, everyday settings wherein individuals may experience it. However, as a result of using field studies, our sample sizes are rather small (*N*s = 83, 74, 67 in our studies), which provides less opportunity to employ extensive statistical analyses and limits the number of variables we could explore.

In each study, we provided participants in the FOMO condition with socially attractive information to induce FOMO and those in the control condition with neutral information to keep their affective states unchanged. While we tried to keep the length of the experimental scenarios and the number of visual illustrations comparable between the two conditions, it is possible that the FOMO condition provided more socially relevant information. One potential direction for future work is to tease apart whether information about alternative events or knowledge that others are involved in these events primarily drives FOMO.

Given the growing interest in and the limited work on FOMO, there are several research ideas that are worth investigating. Our work focuses on exploring FOMO during fleeting experiences such as events and activities. Future research could explore the hypothesized effects in shopping or consumption contexts. In the digital era, consumers can reach information anytime, anywhere, and can therefore easily be distracted from their ongoing experiences. Our conjecture is that our results might apply to consumption domains and hence FOMO may have negative consequences for consumer loyalty.

One idea we considered is whether FOMO may lead to avoidance of information about attractive alternatives in order to limit negative feelings. Our findings imply that exposure to information about existing experiences may not always be beneficial. Learning about attractive alternatives or forgone opportunities may prevent individuals from living in the moment, and therefore people might avoid such information. Selective information search—avoiding discomforting information and attending positive information—has been studied in diverse domains. For example, in finance, investors tend to attend to information more actively when it is in their interest and avoid adverse information [54]. In health services, patients are likely to avoid or delay receiving information that will cause mental discomfort [55]. People often avoid information opposing their views on world events and politics [56]. The basic assumption in this research stream is that individuals tend to defend their views, expectations, and behaviors by avoiding challenging information and attending to information that supports their views. Accordingly, one research topic worth exploring is whether and how FOMO affects selective information search. We would speculate that individuals will try to maintain happiness or contentment by avoiding information that could cause FOMO.

FOMO’s relationship with perceived time sufficiency and time-related stress is yet another research avenue worth exploring. Extant research shows that feeling pressed for time negatively affects subjective well-being [e.g., 57]. Whether FOMO induces perceived time insufficiency—and if it does, what the consequences might be—may provide valuable theoretical and managerial insights.

Finally, future work can further explore variables that foster or help to fight FOMO in different domains, such as examining FOMO as a distractor or focuser. Such work would contribute to the growing stream of research that demonstrates mindfulness’s importance for subjective well-being [58], as well as the effects of distractions during experiences [e.g., 59] and decision-making [e.g., 60].

## Supporting information

S1 Appendix Scenario used in exploratory study. (DOCX)
