# Supplementary material for: A threat to loyalty: Fear of missing out (FOMO) leads to reluctance to repeat current experiences
Source: PLoS One. 2020 Apr 30;15(4):e0232318. doi: 10.1371/journal.pone.0232318 (PMC7192437; doi:10.1371/journal.pone.0232318)
Supplement: S1 Appendix — (DOCX) [file pone.0232318.s001.docx]

**Appendix**

**Scenario used in exploratory study**

The term “FOMO” stands for the “fear of missing out”. FOMO is defined as the anxiety that one feels due to missing out on the pleasing experiences that are going on in one’s environment at any moment. It results from being aware of, but unable to partake in any kind of satisfying event such as a social interaction, a novel or a fun experience, or possession of something favourable.

Today, people are aware of the numerous alternative things that they can be doing at any moment. They have easy access to information about what is going on around them, and what other people are doing, especially through electronic devices and social media. There are many alternative things one can be doing at any moment - alternative events to see, places to visit, movies to watch, social gatherings to attend, etc. - yet not enough resources to involve in all. And people are reminded of these missed out experiences more than ever. Hence, FOMO is the negative affective state that something desirable is happening, we are aware of it, but we are not a part of it.

**Stimuli used in Study 1A**

Identifying information such as country, city and university names that are used in the manipulation scenarios have been disguised for the review process. Copyrighted images that are used in the experimental manipulations have been removed from the manuscript.

**FOMO condition**

[Images have been disguised]

We have conducted a survey with undergraduate students to find out how students are spending this summer. Survey results show that about 80% of the students are currently on holiday at various local and international vacation destinations. Students have indicated [city], [city], [city], and [city] as their most preferred summer vacation places within the country; and the [country], [country], [country], and [country] as their most preferred international summer vacation destinations. Using interrail and work-and-travel programs come up as the most fun and the lowest-cost traveling options especially for far destinations. According to the survey results, students’ main motivations in choosing a vacation spot are to have sea/sand/sun vacations while having fun with friends and family, to explore new places and meet new people, and to do activities that they will not have time to do after graduation.

**Control condition**

[Images have been disguised]

History of [University]

[University] was founded in 1993 as a nonprofit private university in [country]. Since 2000, it has been located in its current campus area in [area location], sprawling over a 62-acre estate. With the Colleges of Social Sciences and Humanities, Administrative Sciences and Economics, Science, Engineering, Law, Nursing, and Medicine, [University] offers 22 undergraduate, 32 graduate, and 18 PhD programs.

[University] is supported by the resources of the internationally renowned [Foundation]. Besides [University], the [Foundation] has invested in several educational institutions such as [Museum], [Library], and [High School].

**Stimuli used in Study 1B**

[Images have been disguised]

**FOMO condition**

Event 1: [Singer] at [venue name]

One of the best pop vocalists in the country [singer], is meeting her fans at [venue name] tonight. Celebrating her 23rd year on stage, she is singing the best of her songs. Be ready for an unforgettable night.

Event 2: [Sponsor] Jazz Festival

It is the night for jazz lovers…the queen of jazz Carmen Lundy is performing at [venue]. She is not only a strong vocalist, but also one of the most creative composers of jazz with more than 100 songs charted in the top 10 on several jazz lists.

Event 3: Coffee Fest

A variety of coffee and novel tastes...different brewing methods, workshops, and a lot more are at Coffee Fest, which takes place in the historic [venue].

Event 4: Open Microfone Stand-up

The best way to end your day. Five talented, cool, and funny comedians put on their stand-up specials. Come join their interactive conversations to catch some big laughs after work.

Event 5: Diary of a Madman

One of Gogol’s greatest short stories put on stage. Following the format of a diary, the story shows the descent of a protagonist into insanity. You’ll be amazed watching a man’s gradual slide from sanity.

Event 6: After-Work Party

Turned into a classic for an evening out. Relaxed atmosphere of unwinding from a day of hard work over a drink or two. This is a great way to enjoy your evening with friends and colleagues.

**Control condition**

Geopolitical Importance of [City]

[Pictures and the detailed description of the city have been disguised]

**Stimuli used in study 1C**

**FOMO condition**

[Images have been disguised]

Event 1: Storytelling Event

This is a night of storytelling. In an entertainment spot in [city], locals are taking the stage to tell five-minute stories around a specific theme announced for the evening. Are you ready to be touched by real experiences?

Event 2: Street Ramblers Event

Great harmony along with talented instrumentals. A bluegrass band steeped in traditional, newgrass, and rock music is performing in downtown [city]. These guys are soon expected to release their albums!

Event 3: Identity Exhibition

This exhibition taking place in a cultural spot of [city] will take you on a tour through identity. It provides experience with hands-on interactive stations that show how your genetics, brain chemistry, and even your friends and social groups help make you who you are.

Event 4: Brewing Event

A local [city] brewery is welcoming customers to have an entertaining night while learning about the entire brewing process, from grain to glass. People get to taste unique and delicious local beers of the area.

Event 5: Selected Works in Exhibition

Selected works of famous national artists are exhibited in a local [city] gallery. The exhibit features new installations, paintings, and sculpture. The objects speak to all audiences.

Event 6: TGIT Event

People will gather with their friends at the TGIT live music event with local performers.

Dancing all night on this TGIT music event is a fun way to prepare for the weekend.

**Control condition**

Brief Information About [City]

[Pictures and the detailed description of the city have been disguised]
